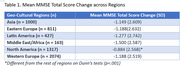# Geo‐cultural Variation in MMSE Score Changes from Screening to Baseline in Alzheimer’s Disease Trials

**DOI:** 10.1002/alz.086988

**Published:** 2025-01-09

**Authors:** Sayaka Machizawa, Erica R. Appleman, Jessica Stenclik, Andrei Iacob, Amanda Hackebeil, Gila Barbati, Jacqueline Massa

**Affiliations:** ^1^ Signant Health, Blue Bell, PA USA; ^2^ Signant Health, Iasi Romania

## Abstract

**Background:**

The Mini‐Mental State Examination (MMSE) is a common screening tool in Alzheimer’s disease (AD) clinical trials. MMSE score inflation at inclusionary visits poses challenges by potentially amplifying placebo responses and complicating the detection of treatment effects. Despite these concerns, prior research (e.g., Echevarria, 2023) has reported instances of MMSE score inflation when it is utilized as an inclusion criterion. Little is known, however, whether this phenomenon is observed universally in AD trials across geo‐cultural regions.

This study examined whether MMSE score changes from Screening to Baseline differ across geo‐cultural regions in global AD trials. The regions compared included North America, Asia, Eastern Europe, Western Europe, Latin America, and the Middle East/Africa.

**Method:**

Data from two multinational Phase 3 AD trials, where MMSE was an inclusionary criterion at Screening but not at Baseline, were analyzed. Both studies used the same MMSE cut‐off for the inclusionary criterion. All raters underwent uniform training and certification for MMSE administration. An enhanced eMMSE scale was employed to minimize rater errors, and a data quality surveillance program included central audio review of eMMSE administration and scoring. Kruskal‐Wallis and Dunn’s tests were used to compare score changes across regions.

**Results:**

Table 1 displays the mean score changes from MMSE Screening to Baseline for each region. A Kruskal‐Wallis H‐test revealed statistically significant heterogeneity in MMSE score changes across regions, χ2 (5) = 16.709, p<.001. Dunn’s tests indicated significantly smaller score changes in North America (p<.001) compared to other regions.

**Conclusions:**

This study observed geo‐cultural variances in MMSE score changes from Screening to Baseline in AD trials. North America demonstrated a significantly smaller score decrement than other regions, indicating potential lesser MMSE score inflation at inclusionary visits. The geo‐cultural differences in MMSE score inflation might reflect cultural impacts on placebo responses. Previous research underscores cultural effects on placebo and nocebo phenomena as cultural factors impact therapeutic expectations, disease perceptions, and treatment interactions (Cundiff‐O'Sullivan et al.). Further investigation is essential to comprehend the factors driving these geo‐cultural disparities in MMSE Screening score inflation and their implications in AD trials.